# Limited long-term changes in tree physiological function despite shifts in forest stand structure following moth (*Lymantria dispar* L.) outbreak and drought

**DOI:** 10.3389/fpls.2026.1776503

**Published:** 2026-05-14

**Authors:** Diane Radwanski, Tomer Duman, Kenneth L. Clark, Dirk W. Vanderklein, Karina V. R. Schäfer

**Affiliations:** 1 Department of Biological Sciences, Rutgers University, Newark, NJ, United States; 2 Department of Biology, University of New Mexico, Albuquerque, NM, United States; 3 USDA Forest Service, Silas Little Experimental Forest, New Lisbon, NJ, United States; 4 Department of Biology, Montclair State University, Montclair, NJ, United States; 5 Department of Earth and Environmental Sciences, Rutgers University, Newark, NJ, United States

**Keywords:** drought, forest disturbance, forest stand structure, leaf gas exchange, water-use efficiency

## Abstract

**Introduction:**

In this study, we investigated forest stand dynamics and leaf gas exchange of co-occurring oak and pine species in an oak-pine forest in the Atlantic Coastal Plain in the Northeastern United States following defoliation by a moth (*Lymantria dispar* L.), and severe drought.

**Methods:**

We used forest stand inventory and leaf gas exchange data spanning from 2005-2015 to analyze the effects of drought and defoliation.

**Results and discussion:**

The defoliation and subsequent drought caused great mortality in a selected group of species and moderate mortality in others. Investigating these dynamics on a decadal time scale (2005 – 2015) revealed a shift in species composition and recruitment in this forest. There was no enhancement of resource use efficiency of the remaining forest species. Long-term water use efficiency in leaves as determined through carbon isotopic analysis declined over the study period. Photosynthetic nitrogen use efficiency of pines and oaks, however, remained relatively unchanged for all species over the period investigated, signaling a preservation of photosynthetic resource use. Also, quantum yield, as a proxy for light use efficiency, decreased over the study period for all species investigated signaling higher light availability. However, the leaves are progressively constructed with less carbon, thus still signaling leaf trait changes. Therefore, with a possible change in species dynamics and given the dynamics in resource use efficiency this forest may not be progressing toward recovery to pre-defoliation status, but rather toward a different, homeostatic, condition.

## Introduction

Forests known to sequester carbon could convert to carbon sources more frequently in the future due to rising air temperatures (Harmon et al., 2011), which are allowing destructive insect herbivores to expand their outbreak ranges (Jepsen et al., 2008; Kurz et al., 2008a, 2008; Dymond et al., 2010; Haynes et al., 2014; Raza et al., 2015; Lesk et al., 2017; Linnakoski et al., 2019). In addition, climate change may exacerbate forest pest infestations, as warmer winters allow widespread survival of insects that destroy forests (Lesk et al., 2017). Insect outbreaks of defoliators result in widespread tree defoliation and can result in subsequent mortality (Kurz et al., 2008a; Brown et al., 2012; Piper et al., 2015), although this depends on the severity, frequency, and timing of an outbreak (Eyles et al., 2013; Piper and Fajardo, 2014). Non-host species and forests containing naïve-host populations may be more vulnerable to invasive insect outbreaks as the defoliators expand their range and their phenology mismatches their host (Abbott and Dwyer, 2007; Cudmore et al., 2010; Kausrud et al., 2011; Pureswaran et al., 2015; Quezada García et al., 2015). Understanding forest response to disturbances such as insect outbreaks is therefore critical, as the expected increase in carbon uptake and eventual carbon saturation of forests could be offset by the increasing frequency of these outbreaks, thereby limiting or resetting productivity or leaf area of the forest (Bouriaud et al., 2025).

The spongy moth (*Lymantria dispar* L.) is a destructive insect defoliator that continues to expand its outbreak range throughout the northeastern United States (Campbell and Sloan, 1977; Twery, 1991; Clark et al., 2010; Schäfer et al., 2010, 2014; Clark et al., 2018; Pasquarella et al., 2018) with outbreaks lasting anywhere from 1 to 30 years (Twery, 1991; Ilyinykh et al., 2011). Defoliation by the moth larvae can cause large scale tree mortality, especially of oaks (*Quercus* spp.), and increase vulnerability of surviving trees to other stressors such as fungal infections, more insect attacks or drought (Herrick and Gansner, 1987; Twery, 1991; Capretti and Battisti, 2007; Galiano et al., 2011; Flower and Gonzalez-Meler, 2015). After re-foliation (Schäfer et al., 2014a), and an initial compensatory up-regulation of photosynthesis (Vanderklein and Reich, 1999; Eyles et al., 2011), a decline in forest productivity by repeated disturbance events could be expected (Quirion et al., 2021; Bouriaud et al., 2025). In addition, more defense chemicals, in the case of oaks, tannins, may be produced to avert more defoliator attack (Pearse and Hipp, 2012). However, the effect of tree mortality and the accompanying decline in competition for resources, as well as increased resource abundance, may actually increase productivity of surviving trees and sub-canopy (Gough et al., 2016; Curtis and Gough, 2018). Canopy gaps left by dead overstory trees increase the amount of light penetrating into the lower canopy (Rozendaal and Kobe, 2014), increasing photosynthesis of surviving trees lower in the canopy, and understory species (Gough et al., 2016). Hence, one might expect an increase in canopy light use efficiency if light was a limiting factor (Gough et al., 2016; Curtis and Gough, 2018).

During and following insect infestations, fewer leaves in the canopy could translate into lower overall transpiration and less rainfall interception (Bréda et al., 1995; Clark et al., 2012). Additionally, the combination of mortality and fewer leaves on surviving trees could result in a greater root to leaf area ratio (Kosola et al., 2001). Likewise, mortality and die back could allow reduced belowground competition and therefore lead to greater area for root exploration. These factors could effectively increase soil water content and plant available water supplies (Bréda et al., 1995; Gebhardt et al., 2014; Olivar et al., 2014) such that, if water is a limiting factor, photosynthesis and productivity should increase, reflected by declines in water use efficiency (Zhao et al., 2009) and increased stomatal conductance (Schäfer, 2011). However, these responses could be delayed if tree mortality following defoliation is delayed (Galiano et al., 2011; Schäfer, 2011). In addition, a recent study also cautioned that fragmented forest canopies do not have the thermal buffer to deal with increases in temperature and, thus, the microclimatic change within the canopy can cause further disruption and change in plant community composition (Zellweger et al., 2020).

Spongy moth defoliation can also have other effects, such as reduced carbon allocation to roots leading to severe declines in nitrogen uptake (Kosola et al., 2001), loss of nitrogen through frass transformations (Kagata and Ohgushi, 2011), faster litter decomposition due to fragmentation, or relocation of nitrogen as moth mobility may effectively remove nitrogen from the infested areas. Furthermore, nitrogen in frass that has been returned to the soil is not necessarily available for plant uptake (Christenson et al., 2002; Kagata and Ohgushi, 2011). Even if trees can recover this nitrogen successfully (Russell et al., 2004) nitrogen allocated to leaves may decline in order to reduce leaf palatability and by producing leaf defense chemicals (Feeny, 1970; Piper et al., 2015) at the expense of photosynthesis and growth (Fyllas et al., 2022). Indeed, the importance of survival and defense against future disturbances may directly reduce growth rate (Wiley and Helliker, 2012; Wiley et al., 2013; Piper et al., 2015) following defoliation events since an increased usage of carbohydrate reserves is required in the short term (Twery, 1991) thus potentially causing an investment in long term carbon storage rather than in leaves (Wiley et al., 2013; Piper et al., 2015).

**Figure 1:**
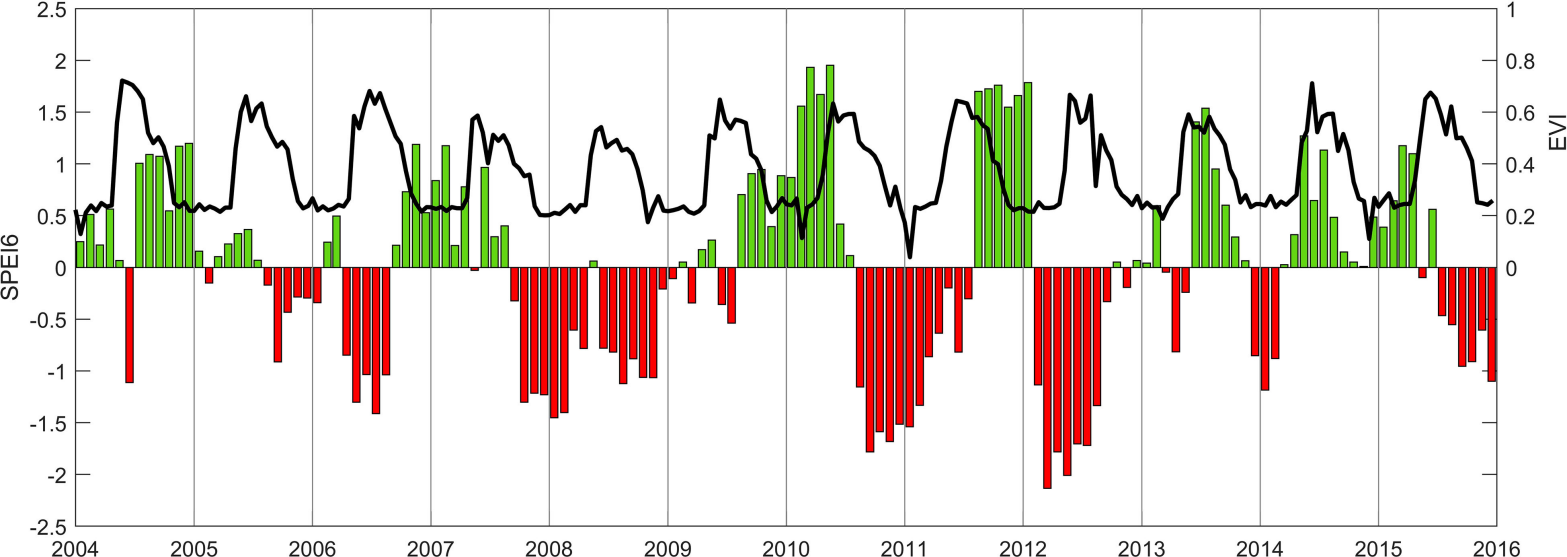
Black line represents the Enhanced Vegetation Index (EVI) and the bars represent the Standardized Precipitation-Evapotranspiration Index (SPEI), whereby red signifies drought and green normal conditions. In winter 2010 and 2011 and in the summer of 2012 severe drought occurred.

While numerous studies have examined plant physiology, including photosynthesis and plant water relations, in response to defoliation (Turnbull et al., 2007; Quentin et al., 2011, 2012; Eyles et al., 2013; McCormick et al., 2013; Jacquet et al., 2014; Schäfer et al., 2014b), many of these studies have examined responses following partial, rather than complete, defoliation events, while also utilizing artificial forms of defoliation (Vanderklein and Reich, 1999; Turnbull et al., 2007; Quentin et al., 2011; Eyles et al., 2013). Others have examined the effects of complete defoliation on photosynthesis utilizing pots in addition to artificial defoliation (McCormick et al., 2013). While the effects of complete defoliation following insect outbreak in natural forest communities has been examined before (Clark et al., 2010; Schäfer et al., 2010; Piper et al., 2015; Clark et al., 2018), these studies focused largely on forest carbon dynamics including forest productivity (Clark et al., 2010; Schäfer et al., 2010; Piper et al., 2015) and ecosystem exchange of CO_2_ (Gough et al., 2008; Clark et al., 2010). Additionally, these parameters were examined over shorter time scales of up to two years following defoliation events (Clark et al., 2010; Schäfer et al., 2010; Piper et al., 2015) or focused on longer term ecosystem level response (Clark et al., 2018). Here, our objective is to examine the long-term effects of leaf eco-physiological function and resource use efficiency after complete natural defoliation caused by a moth outbreak that occurred in 2007 with a partial defoliation in 2008 in an oak/pine forest community in the New Jersey Pine Barrens followed by severe drought events in 2010 and 2011 (cf (Schäfer, 2011; Schäfer et al., 2014a), **Figure 1**). We used leaf level gas exchange measurements, leaf isotopic analysis, and stand inventory and mortality data recorded before and after moth defoliation and drought spanning from 2005 to 2015 to explore long-term forest response in terms of stand structure, leaf gas exchange, and resource use efficiencies. Differences in resource use efficiencies and physiology during drought were highlighted in a comparison of responses between and among two oak groups, the red and the white oak group, and pines (Renninger et al., 2014, 2015), however, these studies did not investigated pre- and post-defoliation and drought responses over a prolonged study period and forest stand composition changes. Thus, this current study aims to synthesize a longer disturbance history of tree physiological function with stand compositional changes. Although the effect of defoliation and drought cannot be disentangled with certainty, the combined effects can be analyzed. We hypothesized that light use efficiency approximated through leaf level quantum yield would increase over the study period (Gough et al., 2016) while water use efficiency should decrease. We further hypothesized that photosynthetic capacity would not be impacted by environmental parameters, stand structure or mortality (disturbance) in this forest ecosystem effectively preserving photosynthetic capabilities as a homeostatic trait (Fernández-De-Uña et al., 2016). As these parameters are critical in long term modeling and projections of future carbon sequestration by forests, they are important to evaluate. The major premise, then, is that photosynthetic capacity is preserved over the long term and constrained within species specific bounds and that leaf area and stomatal conductance are the major ways that trees in this forest ecosystem respond to disturbances, and resource and community shifts (Schäfer, 2011; Clark et al., 2022).

## Materials and methods

### Site description

The research site was located at the Silas Little Experimental Forest which is located within the Pine Barrens of New Jersey (LAT 39.916715, LON -74.597473), the largest continuous forested landscape on the Northeastern U.S. coastal plain. Mean annual temperature was 11.5 °C with an average annual precipitation of 1123 ± 182 mm during the study period from 2005 to 2015 (Schäfer et al., 2014a). Aboveground topography is relatively flat with a mean elevation of 33 m. The soil is a podzol consisting of late Miocene fluvial sediments of the Kirkwood formation underneath Cohansey sandy soil (Tedrow, 1986; Rhodehamel, 1998). The sandy soils have low nutrient content, low water holding capacity, and low cation exchange capacity (Tedrow, 1986; Ehrenfeld et al., 1995, 1997; Markley, 1998; Schäfer, 2011). Dominant tree species in the Silas Little Experimental Forest study site included *Quercus montana* (formerly *prinus*) Willd. (chestnut oak), *Quercus velutina* Lam. (black oak), *Quercus coccinea* Münchh. (scarlet oak), and *Pinus rigida* Mill. (pitch pine) (Mccormick et al., 1973; Lathrop and Kaplan, 2004; Skowronski et al., 2007). Less abundant species included *Quercus alba* L. (white oak) and *Quercus stellata* Wangenh. (post oak) (Skowronski et al., 2007; Clark et al., 2010; Schäfer et al., 2010). For this analysis we grouped the white oak group (*Q. montana*, *Q. alba*) and the red oak group (*Q. velutina*, *Q. coccinea*, *Q. stellata*) together since these are the groupings used by US Forest Service (Moser et al., 2006), their physiologies are similar within the group (Renninger et al., 2014, 2015) and to increase the sample size. Forest understory was characterized by *Gaylussaccia baccata* (Wangenh.) K. Koch (black huckleberry) and *Vaccinium* spp. (blueberry) (Schäfer et al., 2014b). The Pine Barrens is also characterized by a relatively high frequency and intensity of wildfires, and controlled (prescribed) fires (Little and Moore, 1949; Little, 1998). Total spongy moth defoliation occurred in June of 2007 and partial defoliation occurred in 2008, whereby droughts occurred in August of 2006 and a more severe drought in 2010/2011 and 2012 (see also **Figure 1** red bars (Schäfer et al., 2010, 2014)). In the entire Pinelands Reserve, 21% of the area was affected by moth defoliation in 2007 (Skowronski et al., 2014). We reported on differential responses of pine and oak groups previously (Renninger et al., 2014, 2015), but have not investigated the entire record with pre- and post- disturbance data on longer term responses of leaf gas exchange to the stand structural changes.

### Environmental parameters

Environmental measurements included air temperature (*T*_a_, HMP45C Vaisala, Helsinki, Finland)), relative humidity (*RH*, HMP45C Vaisala, Helsinki, Finland), and precipitation (*P*, TE525, Texas Electronics Inc, TX, USA) during the entire study period. Measurements of precipitation throughfall (*P*_T_, TE525, Texas Electronics Inc, TX, USA) were added in 2009, and measurements of soil water content from 0–30 cm (SWC m^3^ m^-3^, CS616, Campbell Scientific, Inc, Logan, UT, USA) were performed in 2006 and 2009-2015. Meteorological variables were collected at 30s intervals and recorded every half-hour using a data logger (CR3000, Campbell Scientific Inc., Logan, UT, USA). Precipitation and air temperature have been used to calculate the Standardized Precipitation Evaporation Index with a moving average of 6 months (SPEI6 (Vicente-Serrano et al., 2010)) and in addition the MODIS (Terra) Enhanced Vegetation Index (EVI) was derived from the area surrounding the research site to represent vegetation dynamics (Didan, 2015).

It has been shown that soil moisture affects canopy gas and energy exchange on timescales between 10 and 100 days, thus, an averaging window of 30 days before leaf gas exchange measurements were done was used to analyze the impact of soil moisture on photosynthetic parameters (Katul et al., 2001). As all photosynthetic parameters are scaled to a standard temperature of 25 °C, the temperature effect is thus eliminated. However, effectively, vapor pressure deficit is driving water losses and thus carbon uptake, and in general influences these on an hourly to daily timescale (Katul et al., 2001), thus daily average VPD on the days of measurements have been used for analysis.

### Forest stand structure

**Figure 2:**
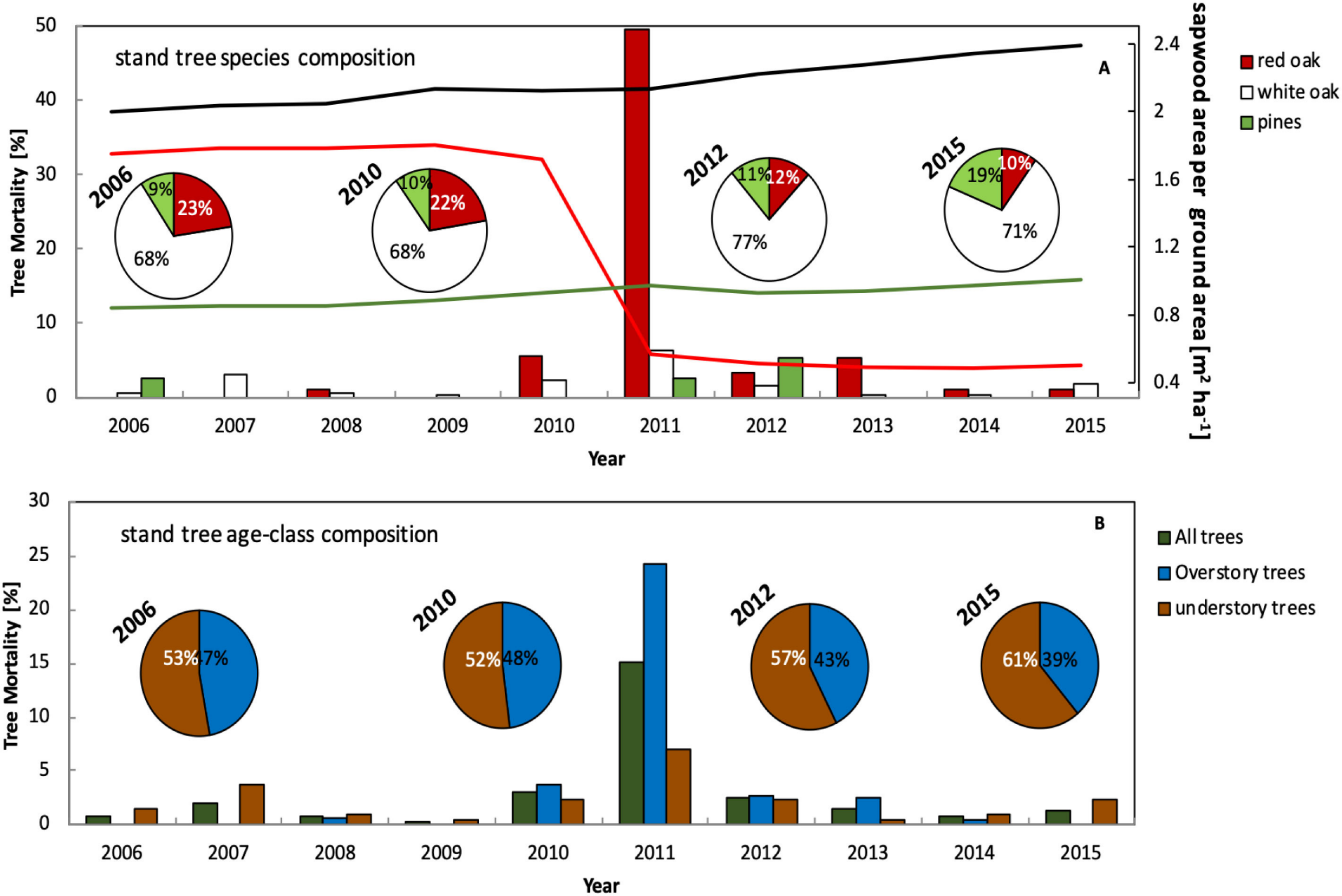
Annual percentage of tree mortality occurring in the Silas Little Experimental Forest from 2006 to 2015. **(A)** the bar chart represents the percentage of trees that died out of all trees in the plot, percentage of overstory (timbersize) trees that died out of the overstory population trees in the plot (dbh ≥ 12.5), and percentage of understory (sapling) trees that died out of the understory population (dbh< 12.5). The pie charts represent the percentage of living trees of all trees (overstory or understory) in 2006, 2010, 2012 and 2015. The lines in the chart represent sapwood are aper unit ground area in m^2^ ha^-1^. **(B)** the bar chart represents the mortality percentage of trees in the plot, separated into white oak group (*Q. prinus* and *Q. alba*), red oak group (*Q. velutina* and *Q. coccinea*) and pines (*P. rigida*). The pie charts represent the percentage of living trees of each species group in 2006, 2010, 2012 and 2015.

The diameter at breast height (dbh) was recorded for the same oaks and pines in the Silas Little Experimental Forest experimental plot each year from 2005 to 2015 for trees with a dbh greater than 2.5 cm within the footprint of an eddy covariance tower about 0.3 ha in size (Schäfer et al., 2010; Clark et al., 2018). The stand inventory also included mortality, and recruitment was recorded when the dbh exceeded 2.5 cm. The percentage of all trees of each species that died was then calculated for each year in the plot to examine trends in tree mortality. Additionally, tree sapling to medium/sawtimber size tree status was noted whereby saplings (understory trees) are greater than 2.5 cm diameter and less than 12.5 cm and medium/sawtimber (overstory, **Figure 2**) size trees were defined as having a minimum dbh of at least 12.5 cm (https://research.fs.usda.gov/programs/fia). Basal area of each species in the plot was calculated assuming the cross-sectional area at breast height to be a circle. The basal area for each species group was summed up and divided by the plot area to derive basal area per unit ground area in m^2^ ha^-1^. Likewise, sapwood area for oaks was calculated as

(1) $$\text{As }({\text{m}}^{2})=\frac{0.0188}{1-e^{-\left(\frac{DBH\left(cm\right)-20.97}{4.791}\right)}}$$

and for pines

(2) $$\text{As }({\text{m}}^{2})=0.3733*{\text{DBH}}^{2.0473}$$

(Renninger et al., 2013, 2015) and summed up for each species/species group and divided by the plot area to derive sapwood area per unit ground area in m^2^ ha^-1^ (see Equations 1, 2).

Aboveground biomass accumulation of understory vegetation (shrubs) was estimated by harvesting 10 clip plots (1.0 m^2^) every year from 2003 to 2015 during the time of peak biomass in mid-summer (Clark et al., 2010, 2018). Fine litterfall was collected during the same time from 10 wire mesh traps (0.42 m^2^). Litterfall samples were then dried at 70 °C at least 48 h, separated into oak and shrub leaves, pine needles, stems, wood, reproductive structures, and frass when present, and weighed.

### Leaf gas exchange measurements

Leaf gas exchange measurements were taken during the last week of each month in 2006 at three canopy levels from May to September (of a total of n=89 for the white oak group, n=140 for the red oak group and n=18 for pines); June, July, and September of 2010 (omitting September n=4 for the white oak group, n= 8 for the red oak group and none for pines); July and August, and September of 2012 (omitting September n=18 for the white oak group, n= 6 for the red oak group and n=3 for pines); July and August of 2013 (n=12 for the white oak group, n= 6 for the red oak group and n=9 for pines); and July, and August of 2014 (n=7 for the white oak group, n= 2 for the red oak group and n=6 for pines), using an infra-red gas analyzer with a broadleaf chamber (LiCor 6400 XT, LiCor Biosciences, Inc., Lincoln, NE, USA). For needle measurements, the same chamber was used and after each measurement, the needles that were enclosed in the chamber, leaf area determined (see also below) and measurements were corrected for respective leaf area (one-sided) in the chamber. In 2006, measurements were taken from three leaves at three different crown heights using a boom lift for each tree, excluding understory trees. Since these intensive measurements are a resource and labor-intensive endeavor it was only done in 2006 for assessment of spatial variation and to be able to evaluate seasonal trends. In 2010, measurements were taken for each tree from two leaves at two different heights, one at breast height and one utilizing a pole pruner reaching a maximum of 5 m height. In 2012, 2013, and 2014 measurements were taken from one leaf or needle bundle for each tree for three to six trees also using the pole pruner. Measurements were made each day from the morning to the afternoon, during peak photosynthetic activity, avoiding the presence of early morning dew on leaves and the natural closure of stomata in the late afternoon. Samples in 2006 were measured *in situ* and following years, samples were taken by cutting branches, located in open areas of the canopy, and then re-cutting them underwater to avoid stomatal closure due to water stress. Leaves of the cut branches that were kept in water, that are a minimum of 1 m length, were measured within 90 minutes after harvest which minimizes loss of photosynthetic capacity and kept the measurement comparable among years (Missik et al., 2021; Davidson et al., 2022).

Assimilation-carbon dioxide response (A/C_i_) curves and assimilation-light response curves were made for each leaf or needle bundle measured with a mean temperature of 25°C +/- 3.9°C, at a flow rate of 500 µmol s^-1^ and humidity between 50-70% in the cuvette. The A/C_i_ curves were constructed by setting a constant light level (photosynthetic photon flux density (PPFD) of 1500 μmol (photons) m^-2^ s^-1^) and altering the CO_2_ concentration in the leaf chamber. Light response curves were constructed by setting a constant CO_2_ concentration of 400 ppm in the leaf chamber while altering light levels (Renninger et al., 2014, 2015).

The data from the A/C_i_ curves were used to calculate the maximum carboxylation rate as limited by Rubisco (V_cmax_; μmol m^-2^ s^-1^), maximum electron-transport-limited carboxylation rate (J_max_; μmol m^-2^ s^-1^), carboxylation rate as limited by triose-phosphate use (TPU; μmol m^-2^ s^-1^), day respiration (R_d_; μmol m^-2^ s^-1^), mesophyll conductance (g_m_; μmol m^-2^ s^-1^ Pa^-1^), carboxylation efficiency (CE; μmol CO_2_ m^-2^ s^-1^ ppm^-1^), and the CO_2_ compensation point (CCP; ppm). Using the A/C_i_ Curve Fitting Utility Version 2007.1 (Sharkey et al., 2007), a best fit model was used to minimize the difference of the sum of square errors between measured and modeled data for the Rubisco-limited, electron-transport-limited, and TPU-limited section of the A/C_i_ curve to estimate V_cmax_, J_max_, and TPU, respectively, as well as R_d_ and g_m_. All values were scaled by the model to a standard leaf temperature of 25 °C. The CE and CCP were calculated by plotting the A/C_i_ data for each measured leaf or needle in Sigmaplot 11.0 (Sysstat Software Inc., San Jose, CA, USA) and fitting a linear regression through the initial, linear section of the A/C_i_ curve. The linear equation of the regression was then used to calculate the slope (CE), and x-intercept (CCP).

The data from light response curves were used to calculate the maximum rate of net photosynthesis (A_max_; μmol CO_2_ m^-2^ s^-1^), quantum yield (Φ; μmol CO_2_ μmol^-1^ photons), dark respiration rate (R_D_; μmol CO_2_ m^-2^ s^-1^), and light compensation point (LCP; μmol photons m^-2^s^-1^) for each measured leaf or needle. As per Lobo et al. (2013), three models were used to best fit the original, observed light response data and the model with the lowest sum of the squares of the errors (SSE) was used. The three models included an exponential curve-fitting model, a non-rectangular hyberbola-fitting model, and a modified non-rectangular hyberbola-fitting model, which utilized equations 8, 6, and 11 in Lobo et al. where the best model was chosen with the best SSE (Webb et al., 1974; Prioul and Chartier, 1977; Ye, 2007; Lobo et al., 2013).

Instantaneous values of net photosynthesis (A_net_; μmol CO_2_ m^-2^ s^-1^), stomatal conductance (g_s_; μmol m^-2^ s^-1^), transpiration (Transp; mmol H_2_O m^-2^ s^-1^), the ratio of intercellular CO_2_ to ambient CO_2_ (C_i_C_a,inst_), photosynthetic water use efficiency (WUE; mmol CO_2_ mmol^-1^ H_2_O), defined as photosynthetic carbon assimilation divided by transpiration, and intrinsic water use efficiency (iWUE; mmol CO_2_ μmol^-1^ H_2_O), defined as photosynthetic assimilation divided by stomatal conductance, were estimated from A/C_i_ and light response curves from points where light levels and CO_2_ concentrations were at PPFD = 1500 μmol m^-2^ s^-1^ and at 400 ppm CO_2_. Altogether, 356 A/C_i_ and light response curves were analyzed over the course of this study.

### Specific leaf weight and isotopic analysis

Leaves and needle bundles that were used for gas exchange measurement were collected, with leaf area determined by scanning with a commercial scanner (EPSON scanner, Epson America Inc) and the image analyzed with imageJ (https://imagej.net). Leaves and needle bundles were then dried at 60 °C for at least 48 hours in a convection oven (Thermo Scientific Precision 3050 Series premium oven, Thermo Fisher Scientific, USA) and weighed to determine specific leaf area (SLA in cm^2^ g^-1^ dry mass). The dried leaves or needles were then ground into a fine powder, placed in tin capsules, and sent out for analysis of leaf C and N concentrations (C and N; %), C:N ratios, isotopic C ratios (δ^13^C), and isotopic N ratios (δ^15^N). The nitrogen per unit leaf area content (N_area_ in g m^-2^) was calculated by dividing N concentration with SLA. The photosynthetic nitrogen use efficiency was calculated from instantaneous net assimilation at 400 ppm external CO_2_ concentration and 1500 µmol m^-2^ s^-1^ photosynthetic photon flux density and dividing by N_area_ (PNUE µmol g^-1^ N s^-1^ (Renninger et al., 2015)).

The 2006 and 2010 leaf samples were analyzed at the Duke Environmental Stable Isotope Laboratory (DEVIL) in Durham, NC, USA. All others were analyzed at University of California’s Davis Stable Isotope center in Davis, CA, USA. Carbon isotopic discrimination (Δ; ‰), isotopic intrinsic water use efficiency (iWUEisotope or short iWUEiso; mmol CO_2_ μmol^-1^ H_2_O), and isotopic ratio of intercellular CO_2_ to ambient CO_2_ (C_i_/C_a,isotope_ or short C_i_/C_a,iso_) were then calculated from the C isotope data utilizing equations from Farquhar et al (Farquhar et al., 1989). The atmospheric ^13^C isotopic composition at the Silas Little Pinelands Experimental Forest has been measured continuously since 2009 by the US Forest Service. The mean growing seasonal δ^13^C and CO_2_ concentrations used to calculate C_i_/C_a, iso_ and iWUE_iso_ were as follows: -8.4 ‰ and 403 ppm in 2009 (and used for 2006 as well), -9.2 ‰ and 396 ppm in 2010, -12.9 ‰ and 407 ppm in 2012, -12.0 ‰ and 410 ppm in 2013 and finally -9.5 ‰ and 410 ppm in 2014, respectively.

### Statistical analysis

**Figure 3:**
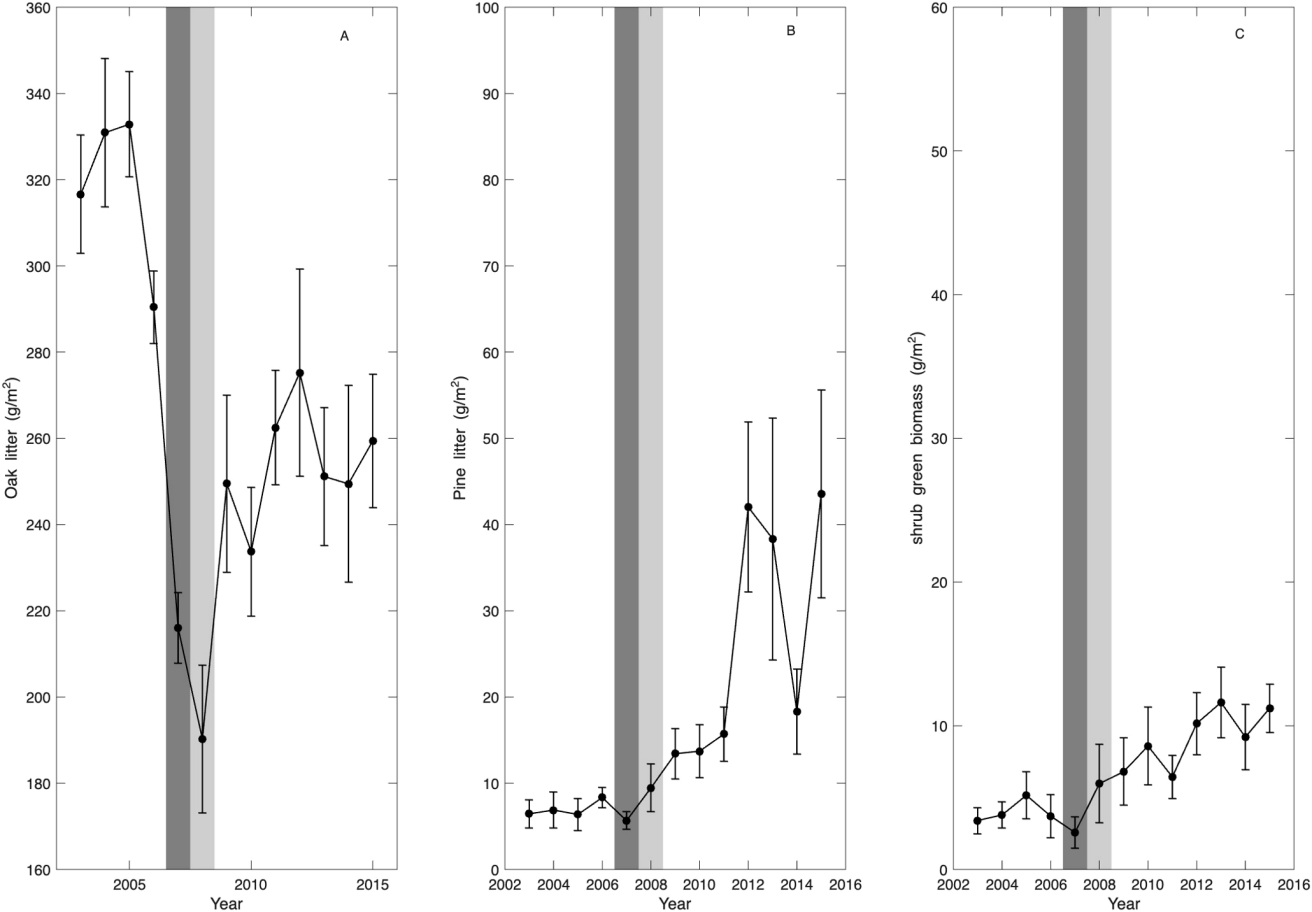
Average annual biomass from litter traps **(A, B)** and clip plots **(C)** in the Silas Little Experimental Forest from 2003 to 2015 for: **(A)** oak leaves, **(B)** pine needles, **(C)** shrub biomass. The vertical bars represent standard error (n=10). The shaded areas represent the moth defoliation in 2007 and partial defoliation 2008 (dark grey) and drought in 2006 and 2010 (light grey).

The data were first examined in 2006 to evaluate differences in canopy position (lower canopy, mid canopy and upper canopy, see **Figure 3**) or season (May through September). The data were evaluated for autocorrelation structure, heteroscedasticity and for outliers (*autocorr* and *parcorr* in Matlab R2025a, The Mathworks. Inc). As the same trees were measured, an autocorrelation was shown for the red oak and white oak group, but not for the pines. Hence, for this dataset a linear mixed effects model with tree ID as random factor and fixed effects of species/species group, month and canopy position (*fitlme* in Matlab) was performed with a species and month interaction. The models with and without interactions were compared and the best model chosen (*compare* in Matlab, see further below). In a further analysis, to compare appropriately between years and species, the same months and canopy position for all years were used, i.e. we used data collected in the lowest canopy position and during the months of June through August during peak growing season while avoiding the shoulder months of May and September in 2006 (resultant n=18 for the white oak group, n= 31 for the red oak group and n=12 for pines). In this dataset, no autocorrelation or partial correlation was detected (α = 0.05) and hence a linear mixed effects model with random intercept and fixed effect of species/species group as a categorical variable and year as a continuous variable (*fitlme* in Matlab), followed by an ANOVA of the model to test whether species or years were different was performed (*anova* in Matlab, R2025a, The Mathworks. Inc). Tree identity was not included in the model. Also here, a model with an interaction of species/species group and year was tested against a model without interaction with a simulated loglikelihood test and the best model chosen. In addition, a contrast (*coefTest* in Matlab) was performed where needed and a α = 0.05 was applied to determine if gas exchange and isotopic means of oak groups and pines differed significantly between years and species groups.

Many leaf traits are thought to be interrelated (Reich, 2014; Keenan and Niinemets, 2016) and thus a Pearson Correlation was performed on the full data set in 2006 for all relevant traits per species and species group and likewise on the dataset from 2010-2014. To discern changes in pre and post defoliation and drought these correlations were done separately for the two time spans.

The underlying assumptions are, that photosynthetic capabilities are not largely impacted by short lived changes in atmospheric or edaphic conditions such as vapor pressure deficit (VPD) or soil moisture (SWC). To ascertain that this was the case, relations of V_cmax_ and Φ to daily mean VPD and monthly mean SWC (Katul et al., 2001) were tested with a linear mixed effects model similar as above, whereby VPD or SWC were the fixed effects and a random intercept with the response variable V_cmax_ or Φ (Matlab, The Mathworks Inc).

## Results

### Environmental conditions and forest stand structure

To contextualize the overall findings with the biotic and abiotic environment, key environmental variables are shown in **Figure 1**. The Standardized Precipitation Evaporation Index with a moving average of 6 months (SPEI6, **Figure 1**, bars) signifies the drought conditions and hence the stress that the trees were subjected to. It combines the atmospheric drought conditions with the soil drought conditions in one parameter and reflects a better measure of the drought stress experienced (Vicente-Serrano et al., 2010). The SPEI shows severe drought conditions in 2010/2011 and again in 2012 (red bars, **Figure 1**). In addition, the enhanced vegetation index (EVI) derived from MODIS (Terra) surrounding the research area reflects the disturbance history with regards to foliage (black line, **Figure 1** (Didan, 2015)). The dip in 2007 and relative lower EVI in 2007 and 2008 reflect the defoliation impact of the area. In the entire Pinelands, 21% of the area was defoliated (Skowronski et al., 2007, 2014), however, the research site had experienced total defoliation in 2007 and partial defoliation in 2008 (Clark et al., 2010).

To explore the forest dynamics, forest stand structure was investigated through an annual stand inventory of the experimental plot, combined with litterfall and clip plots data. Results for tree mortality (**Figure 2A**), mark 2011 as a pivotal year, where 37% of all trees, of which 15% were medium to timber sized trees (‘overstory’), died (**Figure 2B**). Although during 2007 and partially in 2008 the moth defoliation occurred, little change in tree mortality can be seen during those years, as tree mortality remained stable and did not surpass an average annual background rate of 3 to 5% until 2009. After 2010, which included a severe drought event (large negative SPEI values in **Figure 1**), tree mortality increased and peaked in 2011. A year later (2012), tree mortality returned to pre-defoliation levels (**Figure 2A**).

Separating the trees into pines and oak, and distinguishing between trees of the red oak group (*Quercus velutina*, *Q. coccinea*, *Q. stellata*) and white oak group (*Quercus montana* (formerly *prinus*) and *Q. alba* (Renninger et al., 2014)), revealed that most mortality occurred in the red oak group (**Figure 2B**). The stand composition between 2006 and 2010 was stable, and although the plot was mostly dominated by the white oak group, the mortality of 2011 impacted mostly the red oak group population, which comprised 22-23% of the stand composition prior to 2011. The white oak group and the pines were hardly impacted by the mortality of 2011 (**Figure 2B**). The tree inventory also shows that the total number of trees in the experimental plot increased by 10% and that most of the new trees that became established in the experimental plot (with a dbh of at least 2 cm) were first recorded in 2013 and were mostly pines. The changes in tree composition (size and species) are also reflected in sapwood area changes (colored lines **Figure 2B**). The sapwood area per unit ground area drastically decreased for the red oak group after the massive mortality event of 2011, whereby the white oak group and pines only experienced modest losses with gradual rebounding of sapwood area per unit ground area over the years (**Figure 2B**).

Related trends can be seen in the litterfall data, which is a proxy for leaf area (see also **Figure 1**), where in 2007 and 2008 (dark grey shaded area **Figure 3**), the lowest biomass of oak leaves was recorded with about 29% less biomass than the mean biomass of all years investigated here and 40% less than at the beginning of the study period (**Figure 3A**). At the same time, no change was found in pine needle litterfall in 2007 and 2008 (**Figure 3B**). The year 2011 marks an increase in oak litterfall which stayed approximately stable through 2011–2015 but remained about 20% lower than pre-defoliation values (**Figure 3A**). Pine litterfall only slightly increased post-defoliation between 2008 and 2011 (**Figure 3B**). In 2012, a large increase by two and half fold in pine needle litterfall occurred, corresponding to the mortality of oak trees and the appearance of new pines (light grey shaded areas, **Figure 3B**). Thus, the remaining oaks and the pines were able to capitalize on available resources to maintain and enhance growth.

Data from the clip plots show a general increase of shrub biomass, tripling during the study period signaling increased light in the understory (**Figure 3C**). From 2003 to 2006, the change in shrub biomass was small, and increased mainly after the defoliation event of 2007 (**Figure 3C**). During the defoliation event and drought (marked by shaded areas), shrub biomass dropped. Shrub biomass became more variable following defoliation as indicated by the increase in the standard error. This may indicate an increase in heterogeneity in forest stand structure and canopy gaps.

### Leaf gas exchange

**Figure 4:**
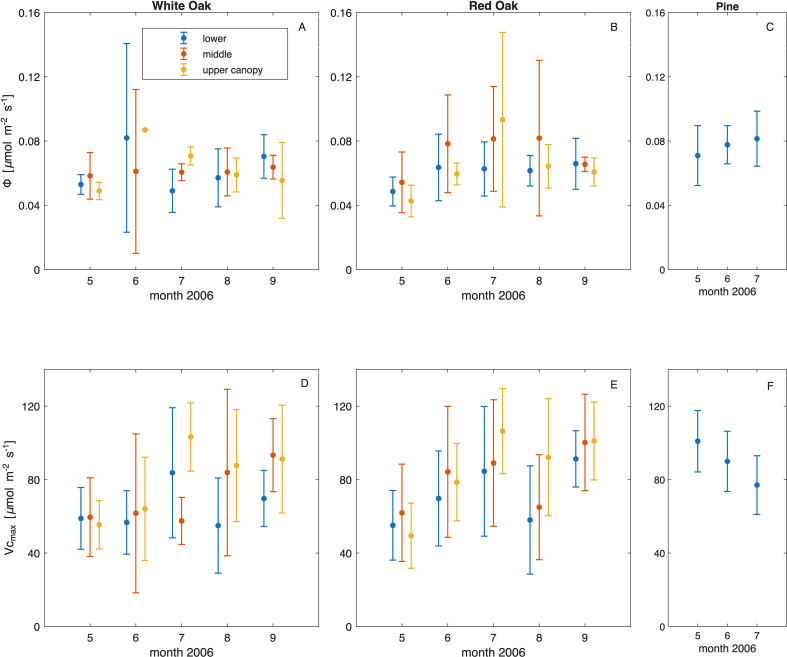
Average monthly quantum yield (Φ in µmol_photons_ m^-2^ s^-1^) in 2006 (top panels, panel **(A–C)** by height (blue: lower canopy, red: mid- canopy, yellow: – upper canopy) and average monthly carboxylation capacity (V_cmax_ in µmol m^-2^ s^-1^ scaled to 25 °C, panel **(D–F)**, lower panels, for white oak group **(A, D)**, red oak group **(B, E)** and pine **(C, F)** with standard deviation of the mean.

In an intensive data collection campaign in 2006, the data were utilized to test whether time of year or leaf canopy position influenced photosynthetic capabilities, thus leaves from different heights were measured throughout the growing season (May to September). Time of year and position in the crown had a discernible effect on quantum yield (Φ) and V_cmax_ for either one of the oak groups at the beginning and the end of the growing season. In **Figure 4** we show the values of Φ (**Figures 4A–C**) and V_cmax_ (**Figures 3D–F**) per canopy position during the 2006 growing season for the white and red oak group and pines, respectively. The data showed only small changes in Φ and V_cmax_ over the tree height range. Comparing month to month values, data collected in May tended to produce lower values of Φ and V_cmax_ for oaks. However, the changes in the monthly averaged values (0.04-0.08 for Φ and 60–90 for V_cmax_) are lower than the maximum range of measured values (0.01-0.2 for Φ and 25–140 for V_cmax_). For Φ, the months were significantly different (F_1,203_ = 8.71, p = 0.003), but not the canopy position (F_1,203_ = 1.34, p = 0.24). Also, the species groups/species were significantly different (F_2,203_ = 6.75, p = 0.001). Whereby May displayed on average a 29% lower Φ for the red and white oak group and only 10% lower for the pines compared to June. Likewise, peak Φ was June for the white oak group, and July for the red oak group and the pines.

For V_cmax_, the months were not significantly different (*anova*, F_1, 217_ = 0.45, p = 0.49), nor the canopy position (*anova*, F_1,217_ = 1.48, p = 0.22), but there was a significant month and canopy position interaction (*anova*, F_1,217_ = 4.50, p = 0.03). The species/species groups were significantly different throughout 2006 (*anova*, F_2,217_ = 3.10, p = 0.04), whereby the pines differed from the white oak group (*coefTest* F_1,217_ = 6.06, p = 0.01) but the white oak group were not different from the red oak group (*coefTest* F_1,217_ = 2.42, p =0.11) highlighting the differences among leaf habit. The shoulder seasons – spring and fall – encounter the largest changes and variations in leaf photosynthetic capabilities (**Figure 4D**, month – canopy position interaction), thus, to be consistent and comparable between 2006 and the data collected in subsequent years, only June, July and August data measured lower in the canopy in 2006 were compiled and compared with values of the other years (Keenan and Niinemets, 2016).

**Figure 5:**
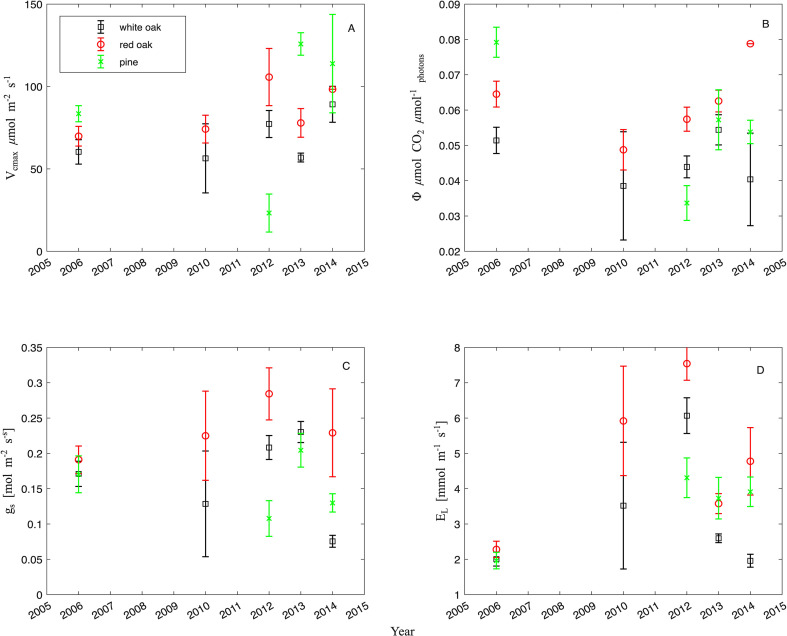
Average gas exchange with standard errors of the mean for the red oak group, the white oak group and pines in the Silas Little Experimental Forest from 2006 to 2014. Abbreviations are as follows: **(A)** maximum carboxylation rate as limited by Rubisco (V_cmax_ in µmol CO_2_ m^-2^ s^-1^); **(B)** stomatal conductance (g_s_ in µmol m^-2^ s^-1^), **(C)** quantum yield (Φ in µmol_photons_ m^-2^ s^-1^) and D) transpiration (E_L_ mmol m^-2^ s^-1^, panel **(D)**).

Generally, photosynthetic parameters varied between years and species. For example, the V_cmax_ of pines had much lower values in 2012 compared to other years (**Figure 5A**). For all years, the oak groups and pines displayed a difference in V_cmax_, with the oak groups having a V_cmax_ more than double that of pines in 2012. This trend reversed in 2013 with pines instead having a V_cmax_ double that of both oak groups. When comparing the red and white oak groups, both had a similar V_cmax_ prior to defoliation in 2006. These values diverged after defoliation, with leaves from the white oak group displaying lower values than those of the red oak group, thus being significantly different (*coefTest* F_1,131_ = 8.56, p = 0.004). Also, the pines were statistically different from the white oak group (*coefTest* F_1,131_ = 11.25, p = 0.001) and the red oak group (*coefTest* F_1,131_ = 8.67, p = 0.003). In addition, the years were significantly different (*anova*, F_1,131_ = 8.86, p = 0.03).

Like V_cmax_, CE, A_net_, and J_max_, had their lowest observed values in 2012 for pines, while the oak groups displayed higher levels (their values, along with values of all other gas exchange parameters, can be found in **Tables 1A–C**). Again, between 2012 and 2013, pines had their largest increase in CE, and A_net_, with values exceeding those of both oak groups (**Tables 1A–C**). Here again, values for the red and white oak groups were similar in 2006 but later diverged with the white oak group displaying lower values than the red oak group resulting in significant differences between species/species groups (*anova* F_2,127_ = 22.88, p< 0.00001 for CE, and *anova* F_2,136_ = 8.65, p< 0.0002 for A_net_). Over the course of the study period, A_net_ varied over the years significantly (*anova* F_1,136_ = 27.72, p< 0.00001) and showed a steady increase for the red oak group, an increase until 2012 followed by a decrease for the white oak group and a fluctuation for the pines (**Tables 1A–C**). In contrast, CE increased for all species/species groups throughout the years with a statistically significant difference between years (*anova* F_1,127_ = 32.29, p< 0.00001).

**Table 1a:** Gas exchange parameters of the white oak group in Silas Little Experimental Forest from 2006 (pre-defoliation) to 2014 (post-defoliation).

Year	2006 (n=89)	2010 (n=4)	2012 (n=18)	2013 (n=12)	2014 (n=7)
V_cmax_	65.6 (9.8)	47.1 (17.5)	77.3 (8.1)	56.9 (2.7)	89.2 (10.9)
CE	0.05 (0.005)	0.05 (0.015)	0.09 (0.011)	0.06 (0.003)	0.09 (0.009)
CCP	62.8 (2.8)	102.0 (17.2)	55.5 (3.0)	61.6 (3.1)	56.5 (4.3)
R_D_	0.36 (0.07)	0.78 (0.29)	1.24 (0.12)	0.80 (0.15)	0.29 (0.10)
Φ	0.05 (0.004)	0.04 (0.010)	0.04 (0.002)	0.05 (0.004)	0.04 (0.011)
LCP	6.6 (0.9)	15.9 (4.0)	25.8 (3.2)	14.8 (3.2)	6.5 (1.0)
A_net_	9.4 (0.8)	7.6 (3.3)	13.6 (0.4)	12.5 (0.3)	9.2 (0.9)
g_s_	0.17 (0.02)	0.10 (0.06)	0.20 (0.02)	0.23 (0.01)	0.07 (0.01)
Transp	2.0 (0.27)	2.8 (1.39)	6.0 (0.44)	2.6 (0.12)	1.9 (0.18)
C_i_C_a,inst_	0.69 (0.02)	0.56 (0.04)	0.64 (0.02)	0.72 (0.01)	0.43 (0.03)
WUE	5.1 (0.40)	3.1 (0.25)	2.4 (0.18)	4.9 (0.18)	4.7 (0.27)
iWUE	63.8 (5.2)	95.4 (11.3)	71.1 (3.9)	57.1 (2.1)	125.9 (7.7)

Standard error of the mean in parenthesis. Sample size in parenthesis per year.

**Table 1b:** Gas exchange parameters of the red oak group in Silas Little Experimental Forest from 2006 (pre-defoliation) to 2014 (post-defoliation).

Year	2006 (n=140)	2010 (n=8)	2012 (n=6)	2013 (n=6)	2014 (n=2)
V_cmax_	73.0 (7.9)	71.4 (5.8)	113.6 (10.1)	77.9 (8.7)	98.4 (0.0)
CE	0.06 (0.005)	0.06 (0.009)	0.14 (0.017)	0.07 (0.003)	0.10 (0.000)
CCP	59.1 (1.4)	101.7 (12.3)	89.7 (11.2)	84.8 (21.1)	47.7 (0.0)
R_D_	0.58 (0.09)	1.09 (0.20)	1.43 (0.17)	0.88 (0.09)	0.95 (0.0)
Φ	0.06 (0.003)	0.05 (0.004)	0.05 (0.003)	0.06 (0.004)	0.07 (0.0)
LCP	9.0 (1.2)	23.4 (6.2)	28.4 (4.0)	14.9 (2.1)	12.6 (0.0)
A_net_	10.2 (0.8)	10.3 (1.8)	14.0 (0.6)	14.6 (0.4)	17.5 (0.55)
g_s_	0.18 (0.03)	0.18 (0.04)	0.20 (0.03)	0.40 (0.04)	0.22 (0.06)
Transp	2.3 (0.31)	4.8 (1.08)	5.7 (0.57)	3.6 (0.28)	4.8 (0.95)
C_i_C_a,inst_	0.67 (0.02)	0.62 (0.03)	0.55 (0.03)	0.80 (0.01)	0.60 (0.08)
WUE	5.3 (0.38)	2.5 (0.19)	2.8 (0.24)	4.3 (0.31)	3.8 (0.64)
iWUE	68.0 (4.3)	76.6 (8.1)	91.7 (9.5)	39.5 (3.6)	82.2 (19.9)

Standard error of the mean in parenthesis.

**Table 1c:** Gas exchange parameters of pines in Silas Little Experimental Forest from 2006 (pre-defoliation) to 2014 (post-defoliation).

Year	2006 (n=18)	2012 (n=3)	2013 (n=9)	2014 (n=6)
V_cmax_	77.0 (6.5)	23.2 (11.5)	125.8 (6.0)	113.9 (29.9)
CE	0.10 (0.01)	0.05 (0.009)	0.16 (0.019)	0.20 (0.046)
CCP	63.6 (6.7)	18.5 (0.0)	88.9 (5.6)	59.6 (7.7)
R_D_	0.5 (0.08)	0.52 (0.08)	0.70 (0.17)	0.76 (0.22)
Φ	0.08 (0.008)	0.03 (0.005)	0.05 (0.009)	0.05 (0.003)
LCP	6.6 (0.8)	16.5 (3.7)	12.9 (3.0)	14.9 (3.6)
A_net_	14.6 (2.11)	8.8 (1.25)	18.1 (1.1)	14.9 (0.9)
g_s_	0.21 (0.04)	0.11 (0.02)	0.20 (0.02)	0.12 (0.01)
Transp	2.0 (0.48)	4.3 (0.56)	3.7 (0.55)	3.9 (0.41)
C_i_C_a,inst_	0.65 (0.02)	0.58 (0.05)	0.57 (0.03)	0.45 (0.04)
WUE	8.3 (1.08)	2.1 (0.16)	5.9 (0.88)	4.1 (0.32)
iWUE	76.6 (5.8)	88.6 (11.6)	97.4 (8.9)	124.8 (19.9)

Values are means and standard errors in parenthesis, and are defined as follows: maximum carboxylation rate as limited by Rubisco (V_cmax_; μmol m^-2^ s^-1^); carboxylation efficiency (CE, μmol CO_2_ m^-2^ s^-1^ ppm^-1^); CO_2_ compensation point (CCP, ppm); dark respiration rate (R_D_, μmol CO_2_ m^-2^ s^-1^); quantum yield (Φ, μmol CO_2_ μmol^-1^ photons); light compensation point (LCP, μmol photons m^-2^ s^-1^); net photosynthesis (A_net_, μmol CO_2_ m^-2^ s^-1^); stomatal conductance (g_s_, μmol m^-2^ s^-1^); transpiration (Transp, mmol H_2_O m^-2^ s^-1^); instantaneous C_i_/C_ainst_, ratio of intercellular CO_2_ to ambient CO_2_; instantaneous water use efficiency (WUE, μmol CO_2_ mmol^-1^ H_2_O); intrinsic water use efficiency (iWUE, μmol CO_2_ mmol^-1^ H_2_O).

Standard error of the mean in parenthesis.

Although, as originally hypothesized, one may have expected an increase in light use efficiency or quantum yield, surprisingly, quantum yield (Φ) either increased (red oak group), decreased (pines) or stayed similar over time (white oak group) with a slight rebound at the end of the study (see **Figure 5B**), hence the photosynthetic utilization of light varied significantly over time (*anova* F_1,124_ = 8.07, p = 0.005) and by species/species group (*anova* F_2,124_ = 8.08, p = 0.0005).

Stomatal conductance which modulates environmental conditions (g_s_, **Figure 5C**) showed significant changes from 2006 to 2012 for both pines and both oak groups. In 2013, g_s_ reached a peak, and then declined below pre-defoliation levels in 2014 for pines and the white oak group (**Figure 5C**). The red oak group increased in 2010 and 2012 and then declined. Thus, overall significant changes of g_s_ over time were detected (*anova* F_1,136_ = 6.27, p = 0.013) and differences between species/species group (*anova* F_2,136_ = 8.88, p = 0.0002).

Transpiration of the oak groups tripled between 2006 and 2012 (**Figure 5D**) and then decreased by approximately 50% from 2012 to 2013 and remained stable in 2014 (**Figure 5D**). Transpiration of pines was also found to change over time significantly between 2006 and 2013 roughly doubling during that time (**Figure 5D**). Thus, the species/species groups differed significantly from each other in their transpiration (*anova* F_2,136_ = 8.88, p = 0.0018, **Figure 5D**) and over time (*anova* F_1,136_ = 41.56, p< 0.00001).

Other examined gas exchange parameters include TPU, CCP, R_D_, LCP, C_i_C_a,inst_, and WUE, and, though sometimes displaying significant differences, they showed only relatively small changes or no strong patterns of increase or decrease over the study period for the two oak groups and pines (**Tables 1A–C**).

### Resource use efficiency

**Figure 6:**
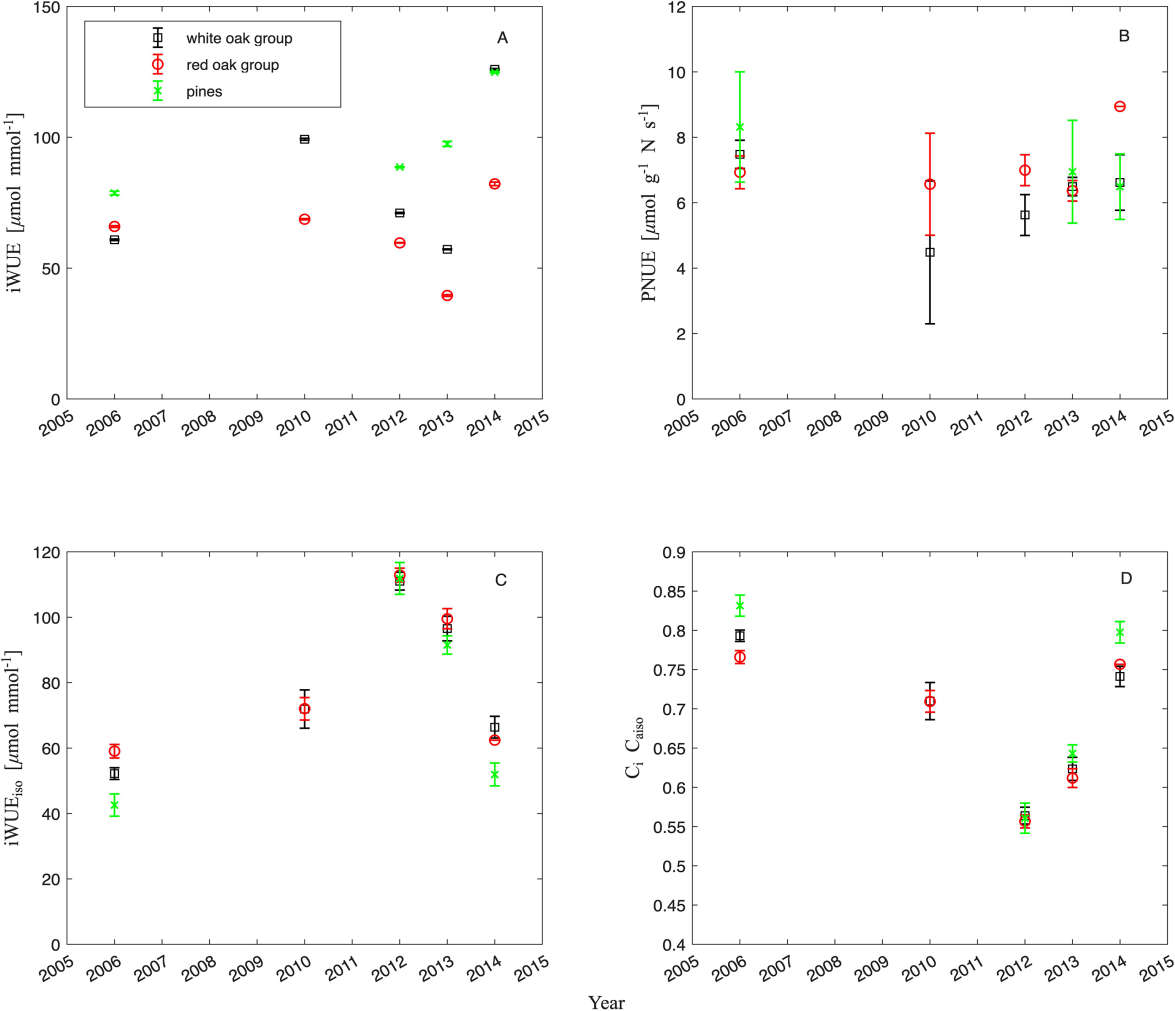
Average intrinsic water use efficiency of the red oak group, the white oak group and pines in the Silas Little Experimental Forest from 2006 to 2014: **(A)** from photosynthetic leaf gas exchange measurements (iWUE in µmol mmol^-1^) and **(C)** from isotopic measurements (iWUE_isotope_ abbreviate iWUE_iso_ in in µmol mmol^-1^); in addition, **(B)** photosynthetic nitrogen use efficiency (PNUE in µmol g^-1^ N s^-1^), and **(D)** isotopically derived internal to external CO_2_ concentration ratio (C_i_/C_aisotope_ abbreviated C_i_/C_aiso_).

The photosynthetic instantaneous intrinsic water use efficiency (iWUE) estimated from leaf gas exchange showed a decrease between 2006 and 2013 for the red oak group, decreasing by roughly 40% during that time, but then rebound in 2014 to 26% higher values than before defoliation (**Figure 6A**). The white oak group followed a similar trend but increased in 2014 even higher at more than double of the pre defoliation levels (**Figure 6A**). Between 2010 and 2012, iWUE levels were similar for the pines, and after 2012 increased by almost double of pre-defoliation levels. The species to year interaction was significant (*anova* F_2,134_ = 7.79, p = 0.0006) and the species/species groups are significantly different from each other (*anova*, F_2,134_ = 7.76, p = 0.0006), however the years are not significantly different (*anova*, F_1,134_ = 1.90, p = 0.16).

The water use efficiency based on ^13^C isotopic data (iWUE_isotope_) showed an increase for the two oak groups and pines from pre- (2006) to post-defoliation levels (2012 and 2013, **Figure 6C**) and then decreased again with 2014 having similar values to pre-defoliation levels (**Figure 6C**). This signals an opposite trend in long-term water use efficiency than for instantaneous intrinsic water use efficiency (iWUE) measurements. Also here, the species/species groups were significantly different from each other (*anova*, F_2,129_ = 7.68, p = 0.0007). However, for these integrated measures of WUE there was a significant effect of year (*anova*, F_1,129_ = 125.9, p< 0.0001) as it was more than doubling by 2012 following a decrease to pre defoliation levels in 2014.

We also examined photosynthetic nitrogen use efficiency (PNUE in µmol g^-1^ N s^-1^, **Figure 6B**), which varied widely with species groups and years, but overall did not display a significant difference between species (*anova*, F_2,104_ = 0.74, p = 0.47) or over the years (*anova*, F_1,104_ = 2.58, p = 0.11), therefore displaying a consistent property among the species and years. Further included in the analysis are derived C_i_/C_a,isotope_ (see **Figure 6D**). However, in contrast to iWUE_isotope_, C_i_/C_a,isotope_ values decreased sharply after the mortality in 2011 and by 2014 had rebounded to pre-defoliation levels displaying significant differences over time (*anova*, F_1,129_ = 121.25, p< 0.0001); again with 2006 and 2014 being similar but different from the intermediate years. Likewise, the species/species groups were significantly different from each other (*anova*, F_2,129_ = 8.00, p = 0.0005). The decline is potentially a combination of a compensatory approach to increased carbon uptake after the mortality created gaps in the canopy and a reduction in g_s_ (see above). Although data for 2007–2009 are not available and conclusions about shorter-term responses to defoliation cannot be made, the existing results show a rebound to pre-defoliation values for all the species investigated. As the isotopic signatures are an integration over the lifetime of the leaf or needle, respectively, it is more commensurate with long term trends than instantaneous measurements.

### Specific leaf weight and chemical analysis

**Figure 7:**
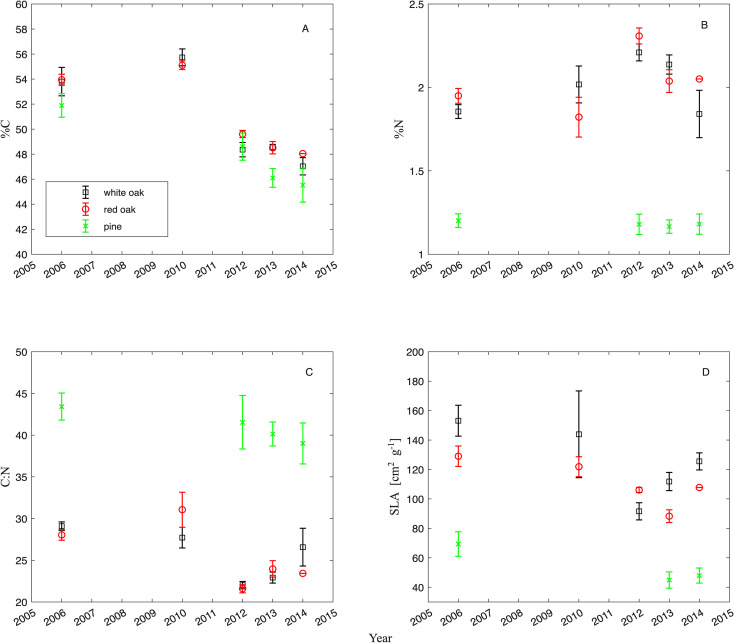
**(A)** Average leaf carbon concentration (C in %), **(B)** leaf nitrogen concentration (N in %), and **(C)** leaf carbon to nitrogen ratio (C/N []), and **(D)** specific leaf area (SLA in cm^2^ g^-1^) from leaves of the white oak group, the red oak group and needles of pines in the Silas Little Experimental Forest from 2006 to 2014.

Further information can be revealed from leaf chemistry data and specific leaf area by examining the carbon and nitrogen content and the morphology of the leaves. Before the moth outbreak, the leaves of all oak species had a 53% carbon content (**Figure 7A**). Following defoliation, leaf carbon content of all species significantly declined after 2011 with pines also displaying a decline in leaf carbon content over the same study period. Leaf carbon content of the pines decreased by roughly 14% from 2006 to 2013 and 2014 (**Figure 7A**). The red and white oak group declined by 10% from 2006 to 2014 in their carbon content, thus there is a significant difference over the years for all species (*anova*, F_1,129_ = 99.66, p< 0.0001) and the species/species groups are different from each other as well (*anova*, F_2,129_ = 6.98, p = 0.0013).

Leaf nitrogen content for all species was found to differ significantly across the study period (*anova*, F_1,129_ = 13.65, p = 0.0003) whereby 2012 stood out with the highest N content in the broad leaves and declining thereafter (**Figure 7B**). Likewise, the species/species groups significantly differed from each other which is not surprising as they are evergreens compared to broadleaf species (*anova*, F_2,129_ = 122.62, p< 0.00001). Leaf nitrogen of the red oak group first declined slightly by 6% from 2006 to 2010, before increasing briefly to above pre-defoliation levels from 2010 to 2012 by 27%, later decreasing again to values more like pre-defoliations levels (**Figure 7B**). During the same period, pines had constant leaf N content hovering at 1.1 to 1.2%. This resulted in the years being significantly different (*anova*, F_1,129_ = 13.65, p = 0.0003).

Leaf C:N ratio differed significantly for both oak groups and pines across the study period (*anova*, F_2,129_ = 165.7, p< 0.0001, **Figure 7C**) as one might expect due to their leaf habit. For pines, leaf C:N steadily declined from 2006 to 2014, decreasing by approximately 18% during that period. The red oak group, however, experienced an increase above pre-defoliation levels in leaf C:N from 2006 to 2010, before declining by approximately 29% by 2012, and then rebounding back some in 2014. The white oak group held steady in 2010, declined in 2012 and increased more in 2014 than the red oak group (**Figure 7C**), thus the C:N ratio changed significantly over time (*anova*, F_1,129_ = 44.2, p< 0.00001). This may indicate that the changes in leaf C:N ratio was largely driven by the change in C over time, as N did not change as much over that same period for all species.

Other examined leaf chemical properties included δ^15^N (see **Tables 2A–C**). δ^15^N was significantly different between species (*anova*, F_2,129_ = 7.67, p = 0.0007), the species/species groups showed a small decrease over the study period (*anova*, F_1,129_ = 13.0, p = 0.0004) and a rebound to pre-defoliation levels in 2014, whereby, again as in C:N, in 2012 the lowest δ^15^N was detected.

**Table 2a:** Isotopic composition and leaf traits of the white oak group in Silas Little Experimental Forest from 2006 to 2014.

Year	2006 (n=89)	2010 (n=4)	2012 (n=18)	2013 (n=12)	2014 (n=7)
C	53.2 (0.75)	55.3 (0.5)	48.3 (0.6)	48.5 (0.2)	47.0 (0.7)
N	1.8 (0.05)	1.9 (0.10)	2.2 (0.04)	2.1 (0.06)	1.8 (0.14)
δ^13^C	-30.2 (0.16)	-29.1 (0.26)	-29.5 (0.22)	-29.9 (0.31)	-30.0 (0.27)
δ^15^N	-4.0 (0.15)	-3.8 (0.29)	-4.4 (0.10)	-4.2 (0.19)	-3.7 (0.18)
C:N	29.1 (0.6)	29.9 (1.5)	22.0 (0.4)	22.9 (0.6)	26.5 (2.2)
C_i_C_a,isotope_	0.80 (0.01)	0.71 (0.01)	0.56 (0.01)	0.62 (0.01)	0.74 (0.01)
iWUE_isotope_	50.4 (1.9)	71.2 (3.0)	111.0 (2.6)	96.5 (3.8)	66.3 (3.3)
SLA	207.1 (59.9)	145.0 (14.8)	91.5 (4.9)	111.8 (6.1)	125.5 (5.8)
PNUE	8.9 (1.5)	3.6 (1.4)	5.6 (0.4)	6.4 (0.3)	6.6 (0.8)

**Table 2b:** Isotopic composition and leaf traits of the red oak group in Silas Little Forest from 2006 to 2014.

Year	2006 (n=140)	2010 (n=8)	2012 (n=6)	2013 (n=6)	2014 (n=2)
C	54.0 (0.5)	52.6 (1.7)	49.5 (0.2)	48.5 (0.5)	48.0 (0.0)
N	1.9 (0.05)	1.7 (0.09)	2.2 (0.04)	2.0 (0.06)	2.0 (0.00)
δ^13^C	-29.5 (0.19)	-29.1 (0.31)	-29.3 (0.13)	-29.6 (0.25)	-30.3 (0.00)
δ^15^N	-3.5 (0.10)	-3.4 (0.27)	-4.3 (0.15)	-3.9 (0.33)	-3.7 (0.00)
C:N	28.1 (0.8)	31.3 (1.5)	21.7 (0.4)	23.9 (0.9)	23.4 (0.0)
C_i_C_a,isotope_	0.76 (0.01)	0.71 (0.01)	0.55 (0.01)	0.61 (0.01)	0.75 (0.00)
iWUE_isotope_	58.7 (2.3)	70.9 (3.7)	112.7 (1.5)	99.5 (3.0)	62.4 (0.0)
SLA	161.8 (31.2)	119.9 (5.2)	105.9 (2.4)	88.2 (4.3)	107.8 (0.0)
PNUE	7.9 (1.3)	5.8 (0.9)	6.8 (0.3)	6.3 (0.3)	8.9 (0.0)

**Table 2c:** Isotopic composition and leaf traits of pines in Silas Little Forest from 2006 to 2014.

Year	2006 (n=18)	2012 (n=3)	2013 (n=9)	2014 (n=6)
C	50.1 (0.7)	48.6 (1.1)	46.0 (0.7)	45.5 (1.2)
N	1.2 (0.04)	1.2 (0.06)	1.2 (0.03)	1.2 (0.05)
δ^13^C	-31.2 (0.24)	-29.4 (0.41)	-30.3 (0.22)	-31.2 (0.26)
δ^15^N	-3.3 (0.24)	-4.2 (0.24)	-3.4 (0.20)	-3.7 (0.28)
C:N	43.3 (1.8)	41.5 (3.2)	40.1 (1.3)	39.0 (2.2)
C_i_C_a,isotope_	0.84 (0.01)	0.56 (0.02)	0.64 (0.01)	0.79 (0.01)
iWUE_isotope_	38.8 (2.8)	111.8 (4.8)	91.5 (2.6)	51.9 (3.2)
SLA	74.7 (16.8)	n.a.	44.9 (3.2)	47.9 (4.6)
PNUE	10.9 (2.5)	n.a.	6.9 (0.7)	46.4 (0.8)

Values are means and standard errors, and are defined as follows: Leaf carbon concentration (C, %); leaf nitrogen concentration (N, %); C:N, leaf carbon to nitrogen ratio; δ^15^N, isotopic nitrogen ratio; C_i_/C_a,isotope_, isotopic ratio of intercellular CO_2_ to ambient CO_2_; iWUE_isotope_, isotopic intrinsic water use efficiency (mmol CO_2_ μmol^-1^ H_2_O), SLA, specific leaf area in cm^2^ g^-1^, photosynthetic nitrogen use efficiency PNUE in µmol g^-1^ N s^-1^

Specific leaf area (SLA) changed significantly over time (*anova*, F_1,113_ = 33.1, p< 0.00001, **Figure 7D**), and the species/species groups were different from each other (*anova*, F_2,129_ = 30.6, p< 0.00001). Specific leaf area of the red oak group declined to an all-time low in 2013 and only slightly recovered in 2014. The white oak group, after an initial decline, rebounded again by 2014 while for the pines, specific leaf area declined slightly from 2006 to 2014. The decline in SLA for the red oak group parallels their declining leaf C content, however, the decline in C content in the white oak group or the pines was not reflected in a concomitant decline in SLA in 2012 and 2014 (**Figure 7D**). All values, along with values of all other isotopic parameters, can be found in **Tables 2A–C** for the two oak groups and pines, respectively.

### Connections and correlations

As one might expect, CE and V_cmax_, as well as V_cmax_ and J_max_, and V_cmax_ and TPU are strongly correlated (R>0.62 pre-, R>0.74 post – defoliation for the red oak group, R>0.45 pre-, R>0.52 post for the white oak group and R>0.33 pre, R>0.99 post for the pines), however, surprisingly, neither N concentration (R = 0.04 pre-, R = 0.25 post for the red oak group, R = -0.05 pre, R=-0.20 post for the white oak group, R = -0.91 pre, R = -0.32 post for the pines) nor N_area_ were strongly correlated with V_cmax_ despite a large range in both (0.9 – 2.9% for N and 10–255 for V_cmax_). A very weak correlation was found between leaf C content and SLA (R = -0.33 pre, R = 0.46 post for red oak group, R = -0.36 pre, R = 0.20 for the white oak group and R=-0.27 pre with post defoliation not enough data for pines) and N and SLA (R = 0.30 pre, R = -0.35 for the red oak group, R = 0.22 pre, R = 0.09 post for the white oak group and R = 0.83 for pines), hence the decline in leaf C content over time cannot be attributed to changes in SLA in the oak groups. Another parameter that experienced alterations over the study period is Φ, which declined for pines and white oaks. Another expected correlation should be between Φ and the maximum photosynthesis derived from the light response curves (R = 0.47 pre, R = 0.71 post for the red oak group, R = 0.27 pre, R = 0.03 post for the white oak group and R = 0.69 pre, R = 0.99 post for the pines), but the correlation was mixed between the two oak groups and the pines.

In order to examine the decline in C:N ratio, the correlation to its constituents may elucidate the underlying driver and the Pearson’s correlation coefficient varied between pre and post defoliation and between the oak groups and pines, whereby a strong negative correlation was found pre and post defoliation for N with C:N for the oak groups and the pines (R = -0.94 pre, R = -0.92 post for red oak group, R = -0.82 pre, R = -0.94 post for the white oak group, R = -0.85 pre, R = -0.82 for pines), but a weaker correlation of C to C:N was found (R = 0.27 pre, R = 0.67 post for the red oak group and R = 0.36 pre, R = -0.06 post for the white oak group, R = 0.69 pre, R = 0.32 post for the pines) with the correlation increasing for the red oak group between pre and post defoliation suggesting that for these species the C:N ratio was driven by both nutrients, whereby for the white oak group and pines nitrogen was likely more driving the change.

A major tenet and underlying assumption are, that photosynthetic capabilities are not largely impacted by short lived changes in atmospheric or edaphic conditions such as vapor pressure deficit or soil moisture. To ascertain that this was the case, relations of V_cmax_ and Φ to daily mean VPD and monthly mean SWC were tested with a linear mixed effects model. Whereby, SWC did not impact V_cmax_ (*anova* F_1,131_ = 2.57, p = 0.11) but Φ (*anova* F_1,122_ = 4.02, p = 0.04), and the species responded significantly different to SWC for V_cmax_ (*anova* F_2,131_ = 5.89, p = 0.003) and Φ (*anova* F_2,122_ = 3.85, p = 0.02). Interestingly, there was also a significant interaction in the response of SWC and the different species to Φ (*anova* F_2,122_ = 4.66, p = 0.01). Likewise, VPD did not appreciably impact the responses of V_cmax_ (*anova* F_1,129_ = 1.25, p = 0.26) and Φ (*anova* F_1,124_ = 1.33, p = 0.25), but the species differed in their response to VPD for V_cmax_ (*anova* F_2,129_ = 10.36, p< 0.0001) and to Φ (*anova* F_2,124_ = 10.29, p< 0.0001). Thus, overall V_cmax_ was preserved with respect to SWC and VPD throughout the study period and Φ was only slightly modulated through SWC.

## Discussion

Forest stand composition changed over the decadal timescale investigated here from a more even mix of red and white oak group species to a more pre-dominantly white oak group species mix with a significant increase in pine saplings. The increased mortality of the red oak group may be explained by their physiology of their acquisitive resource strategy and anisohydry (Renninger et al., 2014; Matheny et al., 2015, 2017; Ratzmann et al., 2019; Asbjornsen et al., 2021; Skelton et al., 2021) at the potential expense of hydraulic failure (McDowell et al., 2008). The increase in pine saplings and understory growth is commensurate with canopy gaps and openings due to downed trees after death (Schäfer, 2011; Stuart-Haëntjens et al., 2015). However, these canopy openings that allowed more light to penetrate and more throughfall to occur did not result in increased light use efficiency across the board as approximated with Φ, but instead resulted in a decline or no change in Φ. Sapwood area per unit ground area of the remaining trees, as a measure for growth rates (**Figure 2B**), increased for the white oak group and pines following mortality but remained relatively constant for the red oak group signaling that the latter group is not yet recovering growth to pre-defoliation levels (**Figure 2B**).

Photosynthesis of the two oak groups, in terms of V_cmax_, J_max_, CE, A_max_, and A_net_, was greatest in 2012, suggesting an increase in resource availability due to reduced competition stemming from high tree mortality in 2011. However, even before mortality set in, oaks exhibited a decline in instantaneous water use efficiency in 2010 compared to pre-defoliation levels (**Tables 1A, B**). This cannot be explained by a reduction in competition due to tree mortality but may instead be attributable to the fact that individual trees would have fewer leaves to support and supply water to, while also experiencing reduced competition from other trees that are also supporting fewer leaves, because the canopies were damaged (Schäfer, 2011). Indeed, others have reported that competition has little effect on isotopic intrinsic water use efficiency (**Figure 6C**) in other oak species (Fernández-De-Uña et al., 2016), albeit we did find an effect over the years. However, our results showed an increase (2013) and then a decline again (in 2014) in isotopic water use efficiency in the two oak groups and pines to levels similar to pre-defoliation (2006), after several years following defoliation. Others have reported finding large increases in water use efficiency following leaf herbivory by leaf miners (Pincebourde et al., 2006), however leaf miners do not consume the leaf but destroy the photosynthetic cells. This resulted from a decrease in stomatal conductance that reduced photosynthesis less so than water use and thus resulted in increased water use efficiency. Most closely resembling and comparing to the isotopic intrinsic water use efficiency are the instantaneous version which indeed, did not show changes over time (**Tables 1A–C**). Moreover, reduced leaf area should allow more water to penetrate the soil and increase the amount of available water to the trees (**Figure 1** green bars (Bréda et al., 1995)). The Pine Barrens is a water-limited environment due to its sandy soils (Tedrow, 1986) such that trees there may carefully regulate water use at the expense of being less efficient with other resources or being restricted from their optimum potential photosynthesis under normal conditions. As such, abundance in water supply should allow trees to release tight regulations on water use, and the increases in transpiration observed in 2010 and 2012 for oaks and pines does suggest that more water was used per unit leaf area. However, SWC *per se* did not affect photosynthetic capabilities in terms of V_cmax_, but did impact Φ in a positive way (positive R).

In this forest, we observed a nearly four-year delay in tree mortality following defoliation (2011 as opposed to 2007 or 2008) and drought (2010/2011) suggesting that certain studies may not detect declines in water use efficiency related to defoliation-drought-induced mortality if trees are examined shortly thereafter (Pincebourde et al., 2006). Furthermore, studies that examine partial defoliation (Pincebourde et al., 2006) are less likely to document mortality since the severity of impact relies on defoliation intensity (Piper and Fajardo, 2014) as well as length of study period, such that certain species that have experienced complete defoliations may still not exhibit any mortality even years after the defoliation event (Piper and Fajardo, 2014). Moth defoliation does not necessarily lead directly to immediate mortality (Gottschalk et al., 1998), but a severe defoliation event does make trees more vulnerable to other stresses (Ojeda et al., 2007) including drought (Schäfer et al., 2014a) or fungal attack which may ultimately cause future death (Fei et al., 2019; Canelles et al., 2021). Responses to either drought or defoliation is also modulated by nutritional status of the trees but they are both deteriorating the health of the trees (Toïgo et al., 2020). Therefore, the timing of these events will vary such that mortality could be delayed even further than observed here (Man et al., 2008) so that even studies examining trees well after defoliation events occur may not detect any changes in function related to defoliation-induced mortality. Limited evidence may suggest trees becoming more resistant to drought after defoliation that could fend off mortality (Itter et al., 2019) but in general more drought induced mortality is expected with exacerbating climate change (Breshears et al., 2005) especially accounting for legacy effects after repeated droughts (Ewers et al., 1999; Guada et al., 2016; Itter et al., 2019) or additional stressors such as defoliation or insect attack (Quentin et al., 2012; Toïgo et al., 2020).

A severe drought occurred in 2010/2011 in the Pine Barrens, which may explain why tree mortality was highest in 2011 (**Figure 1**, **Figures 2A, B**). The overall decline in instantaneous water use efficiency to levels below that of pre-defoliation levels (**Tables 1A–C**), could be explained by a reduction in competition for water amongst surviving trees after large scale mortality. Therefore, the overall decline in water use efficiency observed here over time, could be explained by a release from water restrictions due to lower demand and greater supply, if not by reduced competition for water, light and nutrients. The recovery in intrinsic water use efficiency, which integrates atmospheric conditions, of both oak groups and pines suggests that trees have returned to normal levels of daily photosynthetic water conservation (**Figure 6A** and iWUE_isotope_ **Figure 6C**). The difference in trends between photosynthetic and isotopic water use efficiency may indicate an underlying shift in dynamics between the effects of water and nutrient supply. This may be an indication of a stage in recovery in this forest, since the initial increase in resource availability stemming from tree mortality that is fueling regrowth should restore normal levels of competition and therefore resource access. This is corroborated with steady sapwood area growth in the white oak group and the pines (**Figure 2B**) suggesting a trajectory of recovery, albeit the stand level CO_2_ exchange has yet not rebounded to pre-defoliation levels (Clark et al., 2014, 2018).

The dynamics of water use efficiency are not reflected in the photosynthetic N use efficiency (PNUE, **Figure 6B**), whereby N in the leaves (**Figure 7B**), and PNUE were relatively constant over the decade of investigation suggesting conservation of nutrient use efficiency. Large variability among species in photosynthetic responses has been shown in a temperate forest with a gradient in disturbance severity (Stuart-Haëntjens et al., 2015), hence the ability to capitalize on increased resources is more species specific and time scale dependent and not generalizable. On the other hand, maintaining the same or increased photosynthetic capabilities during defoliation as before may be a survival strategy and may help in the process of recovery and compensation.

Mortality increased canopy openings, reduced leaf area (**Figure 4**) and thus Φ, as a measure of light utilization, declined on the leaf level. This is counter to the argument that light utilization would increase with more light penetrating the canopy due to the gaps (Hardiman et al., 2011), but consistent with the physiological response to sun leaves (McMillen and McClendon, 1983; Jurik et al., 1988; Naidu and Delucia, 1998; Niinemets, 2023). This forest has seen an increasing productivity for the white oak group and the pines, respectively (steeper slopes **Figure 2B**), thus the remaining trees capitalized on more light, water and potentially nutrients.

An interesting aspect was the decline in leaf carbon concentration over the study period in both oak groups and pines and has still not recovered by the end of the study. The initial decline may have been expected as there were fewer leaves and likely fewer resources to devote to leaves in the following years as these resources may have been used in a secondary re-flush for the oaks during the defoliation year itself (Morvan-Bertrand et al., 1999; Schäfer et al., 2014a). However, if competition for light, water and nutrients is reduced, particularly after mortality has occurred, it would be expected that leaf carbon concentration should begin increasing as resource availability increases and especially oak leaves could then invest in tannins for defense (Feeny, 1970; Twery, 1991; Pearse and Hipp, 2012). Leaves should be more heavily invested in C if gaps left by fewer leaves and dead trees allow more sunlight to reach potential leaves, unless light is not a limiting factor. Investment in long term storage in stem tissue instead of investing in leaves could also explain the decline in leaf carbon concentration, as demand from carbohydrate reserves must have increased to allow the re-flush of leaves in 2007 and to some extent in 2008 (Twery, 1991; Jactel et al., 2012; Schäfer et al., 2014a), thus creating a need to rebuild non-structural carbohydrate reserves (Piper and Fajardo, 2014). For oaks at least, this may be a task that requires a longer time due to their deciduous nature, as well as the timing of other future stresses such as the drought in 2010, which could have caused setbacks in rebuilding non-structural carbohydrate reserves. Pines also show this similar long-term decline in leaf carbon, which may indicate a rebuilding of long-term non-structural carbohydrate reserves, but only in the short term (Vanderklein and Reich, 1999). Possibly, the effects of moth defoliation may have been a greater stress on non-structural carbohydrate reserves of pines as compared to oaks, because pines are not the preferred host of these moths (Rossiter, 1987; Davidson et al., 1999, 2001) and because of their evergreen nature (Piper and Fajardo, 2014). Therefore, pines may not be quite as capable of acclimation following complete defoliation as oaks, especially since they retain lower non-structural carbohydrate reserves due to their evergreen nature, thus making a re-flush quite costly in terms of their reserves (Piper and Fajardo, 2014). Given the increasing understanding of the importance of non-structural carbohydrate reserves to tree survival (Hultine et al., 2013; Wiley et al., 2013), trees in the study site may be favoring long-term storage over short-term investment in leaves at the cost of growth (Hultine et al., 2013; Piper et al., 2015), which may explain why photosynthesis has not increased despite that more resources, such as light and water, should be available to surviving trees to increase photosynthetic capabilities. Investment in defense that could reduce frequency of outbreak cycles (Elderd et al., 2013) which consists of thicker cell walls and investment in secondary compounds that are carbon intensive would have increased C content in leaves (Feeny, 1970; Agrawal et al., 2012). Also, the decrease in SLA suggests that the leaves are getting denser per unit leaf area as a morphological adaptation to sun, which is also counter to the findings of decreasing C content (**Figure 7A**). According to an analysis by Poorter and De Jong (1999), lower C content in leaves may indicate higher mineral content, which would hint at “cheaper” leaves, i.e. lower construction cost. This is reflected in a decreasing specific leaf area for the oak groups that has not rebound yet to pre-defoliation levels, albeit the white oak group is in an upward trend and has almost achieved pre-defoliation levels in SLA by the end of the study period (**Figure 7D**). A decrease in SLA is also commensurate with more light and reflects more the anatomy of sun leaves than shade leaves (Givnish, 1988).

The two oak groups displayed an initial decline in leaf nitrogen concentrations over the study period from 2006 to 2010, which can be attributed to many factors. Nitrogen may have been lost through frass and rapid decomposition of leaf fragments, either through insect metabolism that altered the nitrogen into an unusable form (Kagata and Ohgushi, 2011), competition from soil biota (Kuzyakov and Xu, 2013), or simply due to the mobility of the moths. However, if nitrogen was lost, pines should have displayed a similar trend in reduced leaf nitrogen. Instead, pines experienced little change in leaf nitrogen across the study period. However, pines did experience a significant decline in leaf C:N over the study period, suggesting that pines were able to maintain leaf nitrogen concentration throughout the study period, as carbon declined, such that nitrogen was conserved. Furthermore, following mortality in 2011, the oak groups did experience increases in leaf nitrogen in 2012 before declining again by 2014, not only suggesting that nitrogen was likely conserved, but again suggesting that certain resources may have increased due to mortality-induced reductions in competition and that this forest may now be recovering as the declining leaf nitrogen in 2014 may indicate a return to normal levels of competition and resource availability (**Figure 7B**). Additionally, δ^15^N is similar between the oak groups and pines (**Tables 2A–C**), suggesting that both obtained nitrogen from similar sources, such that it is unlikely that pines have been able to maintain nitrogen over the study period because they have different sources than the oak groups. It is interesting to note that δ^15^N was slightly more negative after mortality in 2012, indicating a more biologically processed nitrogen source (Hobbie et al., 1999; Hobbie and Högberg, 2012), but had rebound by 2014 (**Tables 2A–C**). Furthermore, others have found that nitrogen from frass can be both conserved and successfully competed for by trees, perhaps even more so due to an evolutionary development of mutualistic relationships between trees and soil biota regarding nitrogen (Kuzyakov and Xu, 2013). Such evidence may then indicate a strong investment in defense on the part of the oak groups and brings into light important differences between the life histories of pines and oaks. Oaks are these moth’s primary hosts (Twery, 1991) and they may adopt certain defensive strategies such as reducing leaf nitrogen to reduce leaf palatability following defoliation events (Feeny, 1970). Pines, however, are not an ideal host for these moths and were likely only consumed in 2007 because there were not enough oak leaves to support the outbreak in moth larvae population (Rossiter, 1987; Davidson et al., 2001; Schäfer et al., 2010). It is likely, that the leaf habit of evergreens are not as well equipped to withstand repeated defoliation as much as broad leaf species do and hence may be more vulnerable to defoliation (Piper and Fajardo, 2014).

Following mortality in 2011, in contrast to the oak groups, pines experienced the lowest photosynthesis values in terms of V_cmax_, J_max_, CE, A_max_, and A_net_ in 2012 (**Figure 5A**, **Tables 1A–C**), while also experiencing an increase in isotopic water use efficiency (**Figure 6C**). Drought in 2010 had affected the red oak group more as is shown by their mortality (**Figure 2A**). Recent evidence suggests that water availability may be the main driver of forest productivity in the Northeastern US and thus the recovery of this forest may hinge on an even distribution of water throughout the year (Levesque et al., 2017). This would also explain why N, PNUE and V_cmax_ are not altered but WUE and iWUE_iso_ are impacted as not nutrients, but water, is the more limiting factor in this forest. Furthermore, for this particular region a forest model combined with climate forecasts predicted climate and not so much disturbances to play a major role in the forest carbon dynamics (Kretchun et al., 2014). Clearly, recovery of this forest from insect outbreak will be determined to some degree by the differing responses between the oak groups and pines, not only to insect attack, but to future stresses such as drought during recovery.

## Conclusions

In this long-term investigation of tree physiological function after an insect defoliation event in addition to multiple droughts with subsequent mortality, photosynthetic capabilities at the leaf level were partly conserved, were trait-specific and time-scale dependent. Furthermore, resource use efficiency was either not altered (PNUE, **Figure 6B**) or slightly increased (iWUE_isotope_, **Figure 6C**), most likely because of increased water availability due to less competition. Albeit some functions did not experience any changes throughout the time investigated, there are still interesting dynamics that ensued, particularly post-defoliation and mortality, where clearly the system was perturbed and oscillated for a while until settling back to where it was pre-defoliation or at a slightly higher or lower level (see **Figures 5**, **6**, **7**). A more consistent effect ensued with the carbon content of the leaves, that contrary to expectations, dropped instead of increasing, although the leaves have become “denser” for the leaf area supported and hence this warrants further investigation (**Figures 7A, D**). Thus, while resources had increased for surviving trees following defoliation-drought-induced mortality, there will be an eventual return to pre-defoliation levels of resources and tree physiological function as canopy gaps refill and competition is restored.

There is a trajectory of recovery for the white oak group but not for the red oak group even after seven years of the moth outbreak regarding growth (**Figure 2A**) or intrinsic water use efficiency (**Figure 6A**). The recovery process may even be expected to be accelerated for surviving and new trees due to an initial abundance of resources (**Figure 2A**). However, recovery may be slowed due to investment in long-term storage and defense being favored over growth. Additionally, there may be a change in species dynamics in the Pine Barrens, as pines and the white oak group not only appear to be benefitting from a greater water supply (**Figures 5**, **6**) but also have been able to maintain leaf nitrogen and recover SLA (**Figure 7**) sooner following the stress of defoliation. The recovery of the red oak group is still slow to non-existent, which may also be due to increased drought vulnerability, of which pines and the white oak group are less susceptible to (Renninger et al., 2014; Matheny et al., 2017). The case may be that, with a possible change in species dynamics, and given the interesting dynamic between resource use efficiencies, this forest may not be progressing toward recovery to pre-defoliation status in species composition and canopy photosynthesis (Clark et al., 2018), but rather toward a different, yet homeostatic condition. Changes in ecosystem dynamics in response to disturbances like insect outbreak and severe drought may lead to differing carbon sequestration capabilities between our present and future forests and these changes in capability may alter the response of these forests to the effects of climate change (Pugh et al., 2019).

## Data Availability

The raw data supporting the conclusions of this article will be made available by the authors, without undue reservation.
